# Diagnosis of nonexophytic nasopharyngeal lesion with endoscopy-guided core needle biopsy after narrow band imaging

**DOI:** 10.18632/oncotarget.18475

**Published:** 2017-06-14

**Authors:** Min Li, Shenhong Qu, Yangda Qin, Jinlong Lu, Shuilian Yu, Guiping Lan, Jingjin Wen, Yong Yang, Yongfeng Si

**Affiliations:** ^1^ Department of Otolaryngology Head and Neck Tumor, Guangxi Zhuang Autonomous Region People's Hospital, Nanning, China; ^2^ Department of Otolaryngology, Guangxi Zhuang Autonomous Region People's Hospital, Nanning, China; ^3^ Department of Radiology, Guangxi Zhuang Autonomous Region People's Hospital, Nanning, China

**Keywords:** core needle biopsy, nonexophytic nasopharyngeal neoplasms, diagnosis, narrow band imaging, endoscopy

## Abstract

**Background:**

Due to the obstruction of the surrounding structures or stiff mucosa, the primary and recurrent nonexophytic nasopharyngeal carcinoma (NE-NPC) patients are difficult to be diagnosed histologically by traditional forceps biopsy.

**Results:**

All the 15 cases had adequate biopsy for histological diagnosis. There were 5 cases of primary and 7 cases of recurrent NE-NPC, and 3 cases of inflammatory lesion. The histopathological diagnosis was consistent with the follow-up visit. The bleeding quantity during the CNB procedure ranged from 1 to 5 ml (mean 1.93 mL). The pain score during CNB were between 2 and 7 (mean 4.20). There were no serious complications.

**Materials and Methods:**

From April 2009 to March 2016, after conventional white-light and novel narrow-band imaging, nasal endoscope-guided core needle biopsy (CNB) were performed on 15 cases of nonexophytic nasopharyngeal lesion with a semiautomatic biopsy gun.

**Conclusions:**

CNB is able to get adequate biopsy specimens and thus the diagnosis accuracy of CNB is high for NE-NPC. Nasal endoscope-guided CNB is the direct approach with a short distance in the tissue before reaching the tumor. It has the advantages of minimal trauma, short operative time, and no serious complications. It is simple, safe, and worth of application in clinic.

## INTRODUCTION

Nasopharyngeal carcinoma (NPC) is the most common nasopharyngeal malignancy in Southeast Asia [1]. The majority of NPC patients presents in the clinic with an exophytic, ulcerated mass in cavum nasopharyngeum [2]. However, there are still 8.8% of NPC patients have no abnormalities during conventional endoscopic examination, but present with tumor related symptoms, submucosal mass in radiological examination or positive histological results in biopsy [2]. We use the term “nonexophytic nasopharyngeal carcinoma (NE-NPC)” for this subset of NPC. Although most of NPC patients are radiosensitive, about 10–25% of NPC patients still suffer from recurrent disease after definitive radiotherapy [3–5]. Recurrent NPC is also commonly seen in the parapharyngeal space, which is the difficult area for forceps biopsy.

Narrow-band imaging (NBI) is a novel optical technique that enhances the diagnostic sensitivity of endoscopy by using narrow-band width filters in a sequential red-green-blue illumination system. Several studies have shown that NBI is effective in the early detection of head-and-neck squamous cell carcinoma [6–8]. Moreover, NBI endoscopy can improve the sensitivity of detecting mucosal nasopharyngeal neoplasia [9, 10]. Thus, we hypothesize that the diagnosis sensitivity of NE-NPC can also be increased by NBI endoscopy.

The nonexophytic nasopharyngeal lesion is often occult. The mucosa needs to be removed before the suspicious tissue can be acquired by traditional biting cup or punch forceps. However, after radiotherapy, nasopharyngeal mucosa of the NPC patient turned to be stiff. Because of the severe pain and bleeding of the mucosal laceration, the patients cannot tolerate the forceps biopsy procedures. Surgical biopsy under general anesthesia is not advocated for the high cost. Fine-needle aspiration biopsy cannot provide adequate tissue for the histology examination. How to get pathological diagnosis in these patients has been a challenge. In recent years, core needle biopsy (CNB) has been demonstrated to be effective in the diagnosis of various tumors, such as squamous carcinoma and lymphoma [11–13]. Moreover, CNB has been associated with fewer complications and the procedural cost is lower. Percutaneous biopsy of parapharyngeal space lesions with CT guidance were reported [12, 14]. It needs CT scan during the process that cost a lot of money and time. For the long approaches, the risk of the muscles, blood vessels, and nerves injury increased. Nasal meatus are the nature approaches. In the present study, we performed CNB through the nasopharyngeal mucosal under the guidance of nasal endoscopy.

## RESULTS

### Patient characteristics before CNB

**Figure 1:**
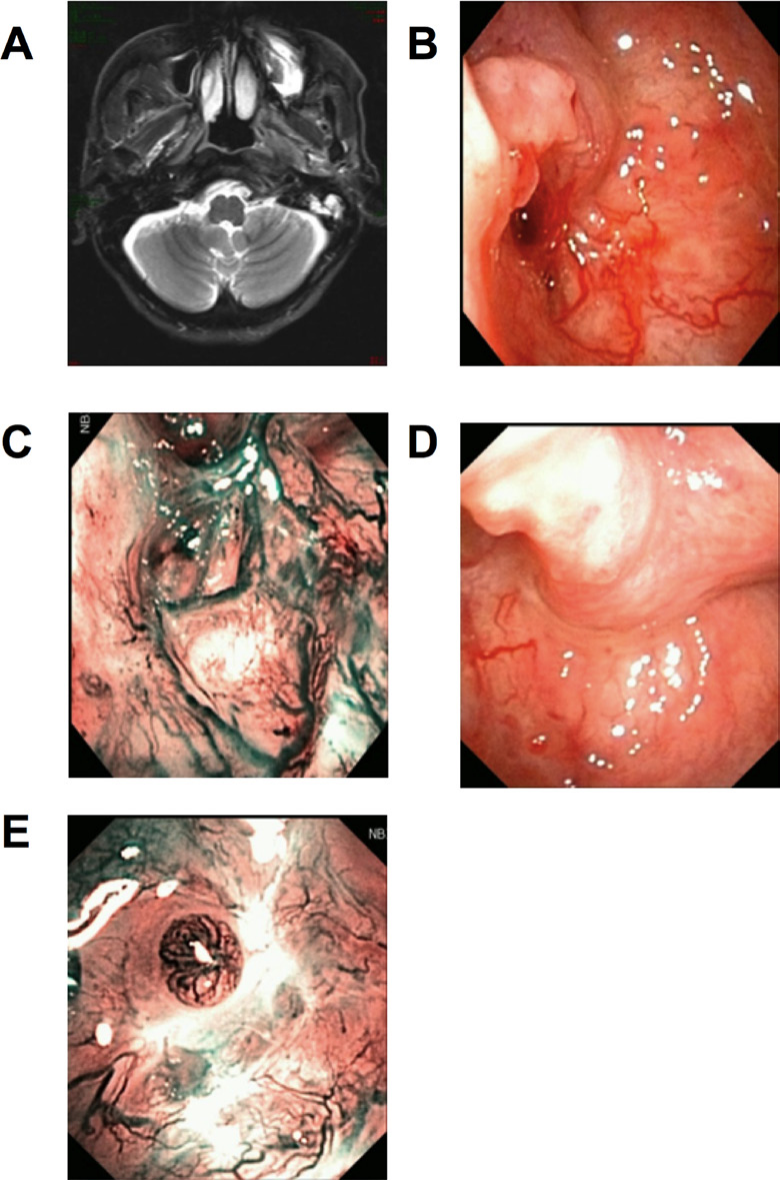
Patients with first diagnosed NPC Head and Neck MRI (T2WI + contrast) scan showed the slightly blunt fossa of Rosenmuller, local bulging mucosa and slightly high signal swollen medial pterygoid and longus capitis on the left side (**A**). Under WL, part of the torus tubarius was missing by forceps biopsy (**B**). Under NBI, mucosa in biopsy area was hyperemia, there was no positive found as brownish spots (**C**). The CNB puncture point with distance and closer view 2 weeks later, under WL and NBI respectively (**D** and **E**).

**Figure 2:**
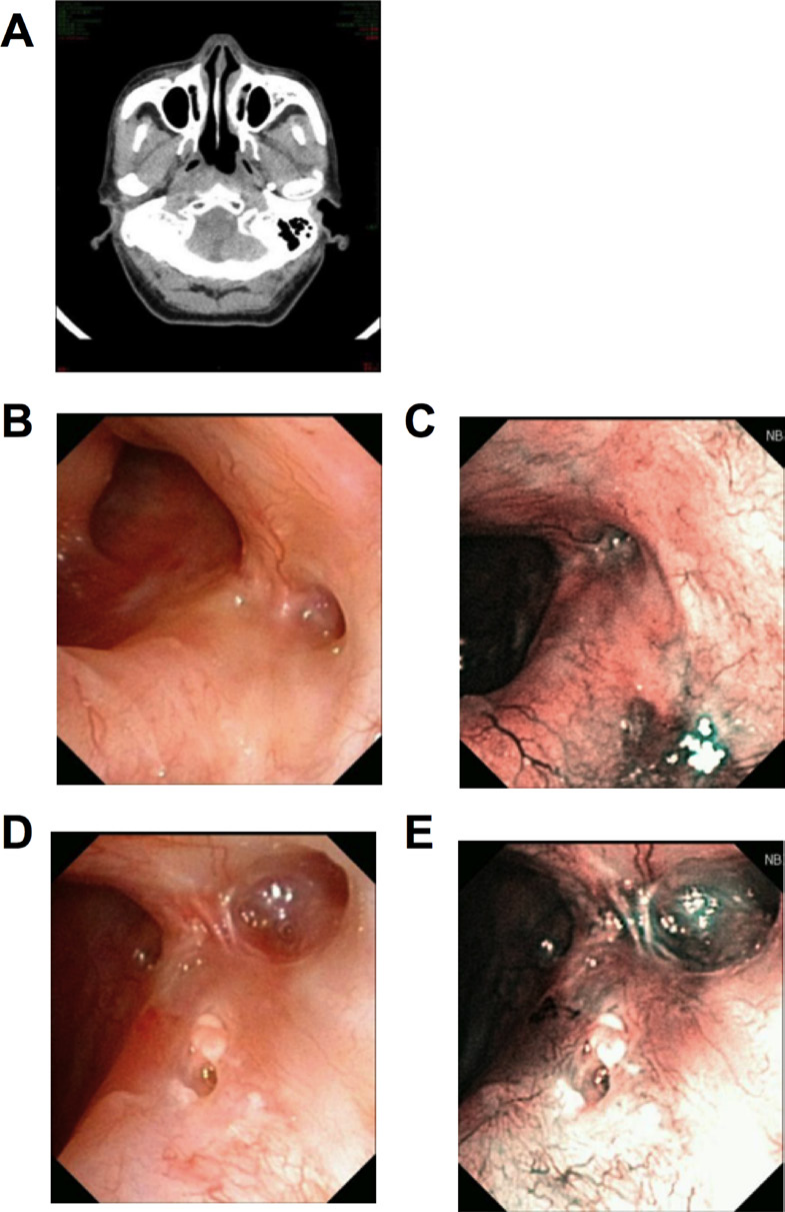
Recurrent patient of NPC CT scan showed swollen longus capitis and parapharyngeal space invasion on the right side (**A**). Under WL, after radiotherapy, nasopharyngeal tissue had local adhesion. Mucosa slight bulging, but smooth, the color and lustre was normal (**B** and **C**). Under NBI, there was no positive found as brownish spots (**D**). The CNB puncture point 1 month later, under WL and NBI respectively (**E** and **F**).

The mean age of the 15 nonexophytic nasopharyngeal lesion patients was 48.93 years (range from 17 to 71 years) (Table 1). Among them, 10 cases of patients had previous history of completed NPC treatment. All patients had normal routine blood examination, liver and kidney function, chest X ray, abdominal B ultrasound and nasopharyngeal contrast enhanced MRI (Figure 1A) or CT scan (Figure 2A), with Karnofsky score equal to or larger than 80. Some patients had PET/CT examinations. The mean minimum diameter of the mass was 2.22 cm (range from 1.2 to 3.7 cm) determined by radiography.

**Table 1:** Clinic features of the non-exophytic nasopharyngeal lesion patients

Case	Age, y	Sex	NPC history	Minimum diameter of the mass	Biopsy times before CNB	Bleed -ing(mL)	NRS of CNB	Oper -ation Time (min)	His -tology	TNM Stage	Treat -ment	Follow -up period (months)
1	32	M	N	2.2	2	1	4	14	UNCC	T3N2M0	CCRT	89
2	43	M	N	3	1	3	5	12	UNCC	T3N1M0	CCRT	56
3	48	F	Y	2.4	0	1	2	9	UNCC	rT4N0M0	GU	Lost
4	51	M	Y	1.5	1	2	4	13	UNCC	rT2N0M0	CCRT	53
5	65	M	Y	1.9	0	1	6	10	UNCC	rT3N1M1	GU	Lost
6	51	M	Y	1.8	0	1	3	9	Inf	—	FU	38
7	40	M	Y	1.6	1	2	5	11	UNCC	rT2N2M1	CCRT	36
8	71	M	N	3.7	1	5	4	12	UNCC	T4N1M0	CCRT	31
9	17	F	N	2.7	3	3	7	15	UNCC	T4N2M0	CCRT	27
10	45	M	Y	2	0	2	6	8	Inf	—	FU	25
11	52	M	Y	1.2	0	3	4	9	UNCC	rT4N2M0	CCRT	20
12	67	M	N	1.6	1	2	3	15	UNCC	T4N3M0	CCRT	13
13	47	M	Y	3	0	1	2	11	UNCC	rT3N0M0	CCRT	11
14	50	F	Y	3.2	0	1	4	8	UNCC	rT3N0M0	CCRT	10
15	55	F	Y	1.5	0	1	4	10	Inf	—	FU	10

CCRT = concurrent chemoradiation therapy, CNB = core needle biopsy, F = female, FU = follow-up, GU = give up, Inf = inflamation, M = male, N = no, NPC = nasopharyngeal carcinoma, NRS = numerical rating scale, r = recurrent, UNCC = undifferentiated non-cornification carcinoma, Y = yes.

### CNB findings

All the nonexophytic nasopharyngeal lesion patients were first examined with white light (WL) endoscope and then NBI. Some patients present an abnormal swelling, but the overlying mucosa appeared otherwise normal. For the 5 cases of primary NE-NPC patients, lymphoid tissues were observed in nasopharyngeal area under WL (Figure 1B), and the lymphoid tissues appeared imbricate arrangement under NBI. For the 7 cases of recurrent NE-NPC patients, due to radiotherapy, local mucosa and lymphoid tissue were atrophy. Local eminence with smooth surface was observed in back/lateral wall of nasopharynx under WL (Figure 1B, 2B). Under NBI, typical abnormal vessels were not found in any cases (Figure 1C, 2C), which indicate all of them are not mucosal NPC.

The mean bleeding amount during the puncture was 1.94 mL (range from 1 to 5 mL) (Table 1). The one to three core biopsies per patient were very well tolerated. The mean pain score of numerical rating scale (NRS) was 4.2 (range from 2 to 7). No complications such as seroma, hemorrhoea and infection in all cases.

### Pathological diagnosis after CNB

CNB provided sufficient tissue for the histopathology diagnostic and no further biopsy was requested in all cases. Among these 15 nonexophytic nasopharyngeal lesion patients, 5 cases of nasopharyngeal undifferentiated non-cornification carcinoma were found by pathology examination. Among the 10 patients with history of completed NPC treatment, 7 cases of recurrent NPC and 3 cases of inflammatory lesion were found. The inflammatory lesions were followed up for 10, 25, and 38 months, respectively, and they had no subsequent diagnosis of NPC during the follow-up. Thus, all the patients had the right pathological diagnosis.

## DISCUSSION

NE-NPC was defined as presenting with normal endoscopic findings, no mass or mucosal ulceration, but with tumor in radiological examination or positive biopsy [1]. The primary tumors usually do not breach the mucosal surface of the cavum nasopharyngeum. Nasoendoscopy and flexible nasopharyngoscopy are sensitive, popular and easy ways to detect exophytic types but not NE-NPC. Head-and-neck MRI, or CT, even 18F-FDG PET is needed to visualize the tumor. Endoscopy is a cheaper way to detect exophytic NPCs. However, for NE-NPCs, a few of them have slight thickening or bulging, while most of them have no obvious change in local mucosa. Under the traditional endoscopy, nasopharyngeal mucosa is smooth, the color and lustre are normal, and with no exophytic neoplasm or ulcer. NBI is a novel optical technique, used “brownish spots” as the typical image pattern for detecting early cancerous lesions in head-and-neck squamous cell carcinoma [6–9]. It has been reported that a more detailed microvascular structure including dilated, tortuous, irregular caliber microvessels could be viewed using NBI closer view [10]. Thus, NBI can help to distinguish nonexophytic lesions from mucosal lesions. The mucosa lesions are easy to get the biopsy. In Kwok's study, 8.8% patients with NPC were NE-NPC [2]. If all the patients had the NBI examination, they may detect that some patients had the mucosa lesions, and the proportion of NE-NPC would be reduced. In our study, we defined histology-proven NE-NPC as: 1) tumor visualized by imaging methods such as MRI, CT or PET-CT; 2) under the endoscopy, nasopharyngeal mucosa is smooth, and there is no exophytic neoplasm or ulcer; 3) no positive results revealed by NBI.

We need enough cytology specimens for immunohistochemical examination to diagnose NPC. Forceps biopsy is good enough for exophytic tumor. For the nonexophytic mass, the mucosa needs to be removed before the suspicious tissue can be acquired by forceps biopsy. However, after radiotherapy, nasopharyngeal mucosa of the NPC patient turned to be stiff, thus biopsy is difficult to be performed with traditional forceps. Some cases even need the surgical biopsy under general anesthesia. Furthermore, traditional forceps cause the mucosal laceration, local tissue injury, bleeding and painful. The patients suffered NE-NPC usually need more than once traditional forceps biopsy, due to negative pathologic results. Because patients often have great fear about the biopsy procedure, they do not accept the biopsy under local anesthesia again. As a result, early diagnosis is often difficult and this may delay the initiation of therapy in patients with malignancy. For those patients had suspected distant metastasis, they didn't receive nasopharyngeal radiotherapy immediately. The trauma caused by biopsy should be as small as possible.

In the present report, tumor history information was acquired, and physical examination, imaging tests and endoscopy examination with NBI were performed before biopsy. Regular and enhanced MRI examination revealed the exact locations of tumor tissues, and their relationship with the neck vessels. In our study, the number of cases with history of NPC is 10, which is more than the cases are first diagnosed. This may due to the fact that the patients with history of NPC had MRI/PET/CT test at each regular follow-up visit. Thus, the lesions can be revealed timely.

The CNB is easy to be accepted by the patients. It takes no more than 15 minutes (Table 1) and is billed at approximately $70 in China. The incision is reduced to a puncture site, and patients experience minimal post-procedure discomfort. Wound infections, seromas, hematomas and nerve injuries are rarely reported after CNB. Fine-needle aspiration biopsy (FNAB) is also used for nonexophytic lesions [15–17]. However, the limitation of the FNAB is that it cannot get enough cytology specimens for immunohistochemical pathological examination. CNB cuts off thin strips of the tumor tissue, meets the requirements of the histopathology diagnosis. This contributes to the low non-diagnostic rate and high sensitivity and specificity of this procedure, which minimizes unnecessary and repeat biopsy.

How to perform the endoscope-guided CNB without clinically significant hemorrhage? The operator need to be familiar with the cross-sectional anatomy of the head and neck region and the location of major vessels, and choose the right needle path. Nonexophytic nasopharyngeal lesions usually occur or invade the parapharyngeal space, the pharyngeal mucosal space, the retropharyngeal space, and the prevertebral space. Those spaces contain vessel, nerves, fat and lymphoid tissue. Compared with percutaneous biopsy of subzygomatic approach (infratemporal, transcondylar, sigmoid notch) under CT guidance, [14] CNB of nasal approach under the guidance of nasal endoscope was easier, cheaper, and less dangerous, and suitable for the lesions in those spaces. The advantages of nasal approach include the natural nasal meatus, clear anatomical marker, direct approach with a short distance in the tissue before reaching the tumor. For reducing the incidence of complications, it is important to puncture mass avoiding internal carotid. There are three ways for the needle passing the nasal cavity: common nasal meatus, middle nasal meatus, and inferior nasal meatus. These methods are safe, because they are limited by osseous structures such as nasal septum, lateral nasal wall, base wall of sphenoid sinus, and the nasal choana. The position of the distance between the anterior nostril and nasal choana, and puncture point shows triangle. It is safe for the puncture approaches inward and downward, because there is no major blood vessels and cranial nerves in this space. The needle upward path is safer, if the base wall of sphenoid sinus is not broken, because the needle path is below the sphenoid sinus, and has a certain distance with internal carotid artery. For the needle outward path, label the tubal torus, the angle is less than 15 degrees, and the injury chance of these blood vessels and nerves in the parapharyngeal space by puncture is significantly decreased. The depth of insertion depends with the tumor size, as possible as the needle groove passing the mass. When the resistance is felt as bone, the puncture maximum depth is reached.

To be noted, CNB is not suitable for suspected vascular or cystic lesions and the enlarged retropharyngeal lymph node, because the procedure would cause bleeding and infections under these situations. The lateral cutting groove of the 18G auto-biopsy gun is 1cm. So, tumor diameter more than 1cm is an indication of CNB. Tumor seeding along the needle track after CNB of a lump in the head and neck cancer is 0.0011%, [18] thus, the chance for needle-path tumor implantation in NE-NPC should be low. The puncture point and the needle-path are all in the region of the following radiation or operation (Figure 1D, 1E, 2D, 2E). There were no patients had recurrent tumor at the puncture point in our study during the follow-up time.

## MATERIALS AND METHODS

### Patient information

From April 2009 to March 2016, 15 patients who had nonexophytic nasopharyngeal lesion were enrolled for CNB examination at the department of otolaryngology head and neck tumor, Guangxi Zhuang Autonomous Region People's Hospital. All patients signed informed consents before biopsy. Seven patients had 1 to 3 times forceps biopsy before CNB, with negative pathologic results, whereas 8 patients had the CNB directly. Before the biopsy procedure, the relationship between parapharyngeal structures and tumors was learned through MRI, CT or PET/CT images. Needle path, direction, and biopsy point were preliminarily designed. The biopsy point was selected at the maximum section of parapharyngeal lesions as much as possible.

### CNB procedure

**Figure 3:**
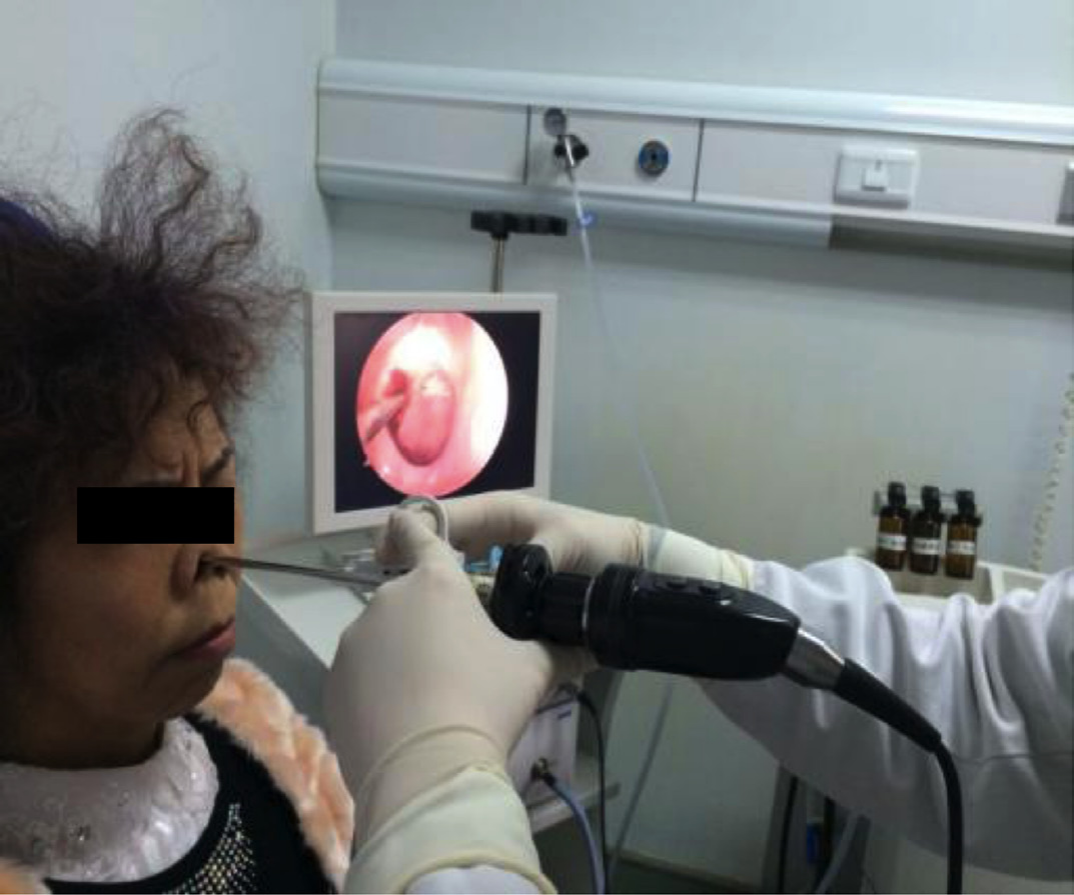
CNB was conducted under the guidance of nasal endoscope

The patients were in a seated position (Figure 3). The nasal cavity of each patient was anesthetized with a spray mix of 1% ephedrine and tetracaine. High performance endoscopic system (VISERA Pro OTV-S7Pro, Olympus Medical Systems, Tokyo, Japan) equipped with the WL mode and NBI mode was introduced in the examination of nasopharynx. Disposable 18-ga semiautomatic needles (18 G × 130 mm, Stericut with a co-axial guide; TSK Laboratory, Tochigi, Japan) were used. Puncture was conducted under the guidance of nasal endoscope, and the core needle arrived at the nasopharynx through nasal meatus. The specimen notch of the inner stylet was set within the target. The puncture was performed as designed. The point, angle and direction were adjusted. The semiautomatic biopsy gun was fired 1 to 3 times to obtain core specimens. The specimens were fixed with 10% formalin and performed histopathology examination. The puncture point was closely observed for 5 minutes to confirm no bleeding or hematoma formation.

All the patients had a follow-up examination with WL and novel NBI, MRI or CT every three months for the first two years after the CNB, and then once half a year. After three years, the examination was performed once a year.

### Numeral rating score and pathology staging

The patients were asked to score the pain they felt during the CNB procedure using NRS [19]. The extent of pain ranges from 0 to 10, where 0 indicates “no pain” and 10 indicates “worst pain imaginable”. The 2002 International Union Against Cancer (UICC) and the American Joint Committee on Cancer (AJCC) TNM system was used for the tumor pathology staging [20].

## CONCLUSIONS

CNB is able to get adequate biopsy specimens, and It can help the patients to obtain histological diagnosis as early as possible. The patients have nonexophytic nasopharyngeal lesions can afford it and bear it. Nasal endoscope-guided CNB is the direct approach with a short distance in the tissue before reaching the tumor. It is a useful alternative way for the conventional nasopharyngeal biopsy, specially for the retropharyngeal and parapharyngeal lesions. It is simple and safe, worth of further clinical study and application.

## Abbreviations

AJCC: the American Joint Committee on Cancer
CNB: endoscopy-guided core needle biopsy
FNAB: fine-needle aspiration biopsy
NBI: narrow-band imaging
NE-NPC: nonexophytic nasopharyngeal carcinoma
NPC: nasopharyngeal carcinoma
NRS: numerical rating score
UICC: the International Union Against Cancer
WL: white light
